# Efficacy of a Short Role-Play Training on Breaking Bad News in the Emergency Department

**DOI:** 10.5811/westjem.2019.8.43441

**Published:** 2019-10-14

**Authors:** Jean-Christophe Servotte, Isabelle Bragard, Demian Szyld, Pauline Van Ngoc, Béatrice Scholtes, Isabelle Van Cauwenberge, Anne-Françoise Donneau, Nadia Dardenne, Manon Goosse, Bruno Pilote, Michèle Guillaume, Alexandre Ghuysen

**Affiliations:** * University of Liège, Department of Public Health Sciences, Liège, Belgium; † University of Liege, Interdisciplinary Medical Simulation Center of Liège, Liège, Belgium; ‡ Institute for Medical Simulation, Center for Medical Simulation, Brigham and Women’s Hospital, Harvard Medical School; § University Hospital Centre of Liège, Department of Emergency Medicine, Liège, Belgium; ¶ Université Laval, Faculté des sciences infirmières, Québec, Canada

## Abstract

**Introduction:**

Breaking bad news (BBN) in the emergency department (ED) represents a challenging and stressful situation for physicians. Many medical students and residents feel stressed and uncomfortable with such situations because of insufficient training. Our randomized controlled study aimed to assess the efficacy of a four-hour BBN simulation-based training on perceived self-efficacy, the BBN process, and communication skills.

**Methods:**

Medical students and residents were randomized into a 160-hour ED clinical rotation without a formal BBN curriculum (control group [CG], n = 31) or a 156-hour ED clinical rotation and a four-hour BBN simulation-based training (training group [TG], n = 37). Both groups were assessed twice: once at the beginning of the rotation (pre-test) and again four weeks later. Assessments included a BBN evaluation via a simulation with two actors playing family members and the completion of a questionnaire on self-efficacy. Two blinded raters assessed the BBN process with the SPIKES (a delivery protocol for delivering bad news) competence form and communication skills with the modified BBN Assessment Schedule.

**Results:**

Group-by-time effects adjusted by study year revealed a significant improvement in TG as compared with CG on self-efficacy (P < 0.001), the BBN process (P < 0.001), and communication skills (P < 0.001). TG showed a significant gain regarding the BBN process (+33.3%, P < 0.001). After the training, students with limited clinical experience prior to the rotation showed BBN performance skills equal to that of students in the CG who had greater clinical experience.

**Conclusion:**

A short BBN simulation-based training can be added to standard clinical rotations. It has the potential to significantly improve self-efficacy, the BBN process, and communication skills.

## INTRODUCTION

Breaking bad news (BBN) is considered to be one of the most important, stressful, and challenging responsibilities of a physician.^1^–^6^ Trainees and experienced physicians alike report being uncomfortable with this task, notably due to a lack of prior training.^6^–^8^ For patients, the acknowledgment of this information and their comprehension and perception are of paramount importance to facilitate their psychological adjustment and a long-term quality relationship with medical caregivers.^9^–^12^

The BBN process has changed drastically over the past decades, moving from a paternalistic medical approach to one of greater patient empowerment, which acknowledges the need for information^13^–^14^ and results in a greater awareness and clearer understanding of their diagnosis and prognosis.^13^ Patients prefer to receive individualized, comprehensive information communicated with warmth and honesty.^15^–^18^ Patient and family expectations regarding the exact content of news have been shown to be highly variable,^13^ making it difficult for healthcare professionals to tailor the information to suit each patient.^19^

Bad news in an emergency department (ED) may consist in announcing that a relative has been admitted to the ED or in sharing with patients or their families news concerning the need for hospitalization or conditions that might lead to a life-threatening situation sooner or later.^20^ BBN in the ED is a particular challenge because the patient is generally meeting the emergency physician (EP) for the first time and neither of them enter into the relationship by choice. A recent survey^21^ revealed that 78.1% of BBN occurred without previous contact between the patient and the physician. Moreover, history taking, diagnosis, and the acknowledgment of bad news are usually accomplished within a very short time frame^22^ during which the physician is confronted with distractions, stress, or time constraints.^23^

EP training in communication skills to notify family members of a patient’s death has been reported to be poor at best,^24^ leading medical students, residents and young physicians to adopt inappropriate communication behaviors,^4^,^25^ which in turn significantly increase their stress levels.^2^ Inappropriate communication behavior does not take into account the needs of patients or their families. Several guidelines have been developed in oncology to help physicians deliver bad news.^4^,^26^–^31^ One of the most widespread BBN protocols is the SPIKES (Setting, Perception, Invitation, Knowledge, Emotions and Summary) protocol.^28^

BBN training in the ED has scarcely been studied to date.^32^ The studies undertaken have included a limited number of participants,^33^–^34^ no validated assessment tools^35^ or control group,^34^,^35^ or were limited to death notification only.^21^,^24^ In this study, we assessed the effects of incorporating a four-hour ED BBN simulation-based training (BBNSBT) on self-efficacy, the BBN process, and communication skills among medical students and junior residents who rotated in the ED. We hypothesized that BBNSBT has the potential to increase self-efficacy, the BBN process, and communication skills.

**Population Health Research Capsule**

What do we already know about this issue?

*A lack of training in breaking bad news (BBN) leads to inappropriate communication behaviors. BBN protocol has been developed to help physicians to deliver bad news*.

What was the research question?

What are the effects of a role-play on the BBN process among students who rotated in the emergency department?

What was the major finding of the study?

*A short BBN simulation-training has the potential to improve the BBN process and communication skills*.

How does this improve population health?

A four-hour, simulation-based training is a good way for trainees to master BBN and better inform the patients and their families on diagnosis and prognosis

## METHODS

The ethics committee approved the study (reference number 2015/235). Only authorized individuals had access to the data and materials. The researchers did not participate in the training program.

### Training Program

#### Control Group

The control group (CG) followed the traditional 160-hour ED rotation. Trainees cared for ED patients under the supervision of EPs. The CG did not receive any formal BBN training.

#### Training Group

The training group (TG) received a traditional 156-hour ED rotation and four hours of BBNSBT.

#### BBNSBT

Participants were split into small groups up to six members. The BBNSBT involved two components: 1) a one-hour theoretical course on BBN, SPIKES and communication skills with a 15-minute video illustrating SPIKES components; and 2) a three-hour simulation including six role-plays. Three participants were included in each role-play (one playing the physician and two playing family members) while the three other participants watched the simulation. Each one took 10–15 minutes plus 20–25 minutes for a debriefing. The debriefings followed the framework for Promoting Excellence and Reflective Learning in Simulation, using the advocacy-inquiry technique.^36^–^40^ The debriefings focused on the SPIKES protocol and effective communication behaviors.

The following steps ensured the consistency of the BBNSBT: 1) the International Nursing Association for Clinical and Simulation Learning (INACSL) Standards of Best Practice for Simulation^SM^^41^,^42^ were used to design the BBNSBT; 2) six experts including psychologists, EPs, and simulation instructors validated the scenarios and simulation design; 3) the same facilitators, a psychologist and an EP trained in BBN and certified as basic simulation instructors conducted training; 4) PowerPoint slides with major theory points accompanied the theoretical part of the BBNSBT; and 5) prewritten scripts were used for the role-play explanations and the debriefings.

### Recruitment

Medical students and first-year residents specializing in emergency medicine (EM) who had recently graduated were included in the study for one academic year, between September 2017–June 2018. A convenience sample was invited to participate in the study. It included medical students (n = 64) following a one-month ED internship and first-year EM residents (n = 9) beginning their first month of internship. Each participant gave his or her signed informed consent on a voluntary basis. Five students did not complete the rotation and were excluded from the study; therefore, a total of 68 participants were included. The TG and the CG had, respectively, 37 and 31 members.

### Study Design

**Figure 1:**
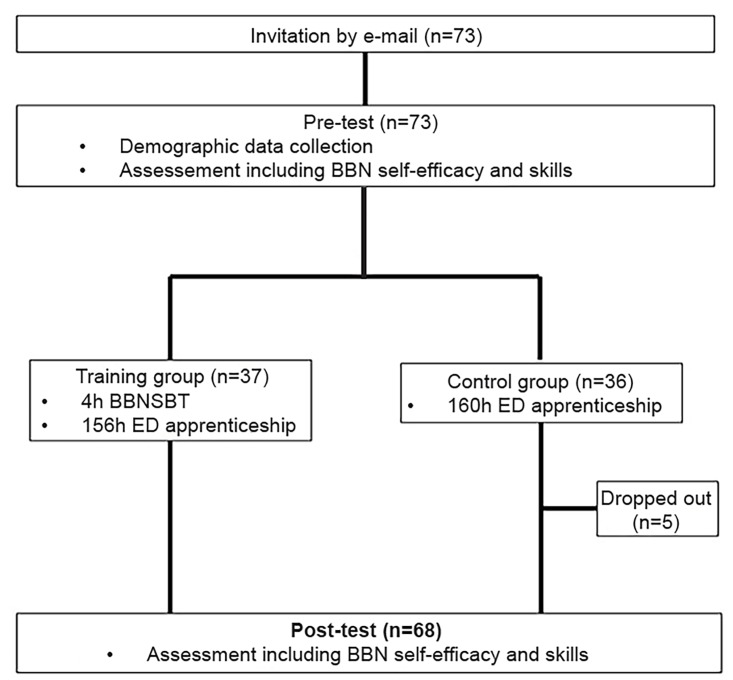
Flowchart of study examining the effect of simulation training on how trainees deliver bad news. *BBN*, breaking bad news; *BBNSBT*, BBN simulation-based training; *h*, hour; *ED*, emergency department.

The feasibility study used cluster randomization to reduce contamination bias.^43^ Each month, a group of 10 to 12 medical students and first-year EM residents was randomly assigned either to the TG or the CG. Demographic data such as gender, age, BBN experience, and study year were collected. During the first week of internship, participants underwent a pre-test of their ED BBN self-efficacy and skills. During the second week, participants assigned to the TG participated in the BBNSBT. Post-testing took place four weeks later and included a self-efficacy and ED BBN assessment (Figure 1).

### Assessment Tools

#### Self-efficacy

We assessed the BBN self-efficacy of participants using a seven-item questionnaire (supplemental material 1 specifically developed for this study, corresponding to seven skills (eg, “To manage your nonverbal communication during the BBN”). Participants rated each item on a Likert scale from 1 (“not at all”) to 5 (“entirely”) in three separate areas: knowledge about the skill; ability to manage the skill; and applying the skill in practice. The experts placed the content validity index of the questionnaire at 0.92.^44^

#### BBN Skills Assessment

BBN skills were assessed in simulation exercises involving two standardized family members played by actors. A randomly selected BBN scenario was used to assess each participant in both pre- and post-test. The scenarios were as follows: 1) a life-threatening situation after a motorcycle accident; 2) a life-threatening cardiogenic shock; and 3) brain damage after a fight. Each trainee performed in one random scenario. The scenarios for the pre-test and the post-test were different in order to avoid memorization bias. The BBN skills assessments were video recorded and anonymized.

Two blinded raters assessed participants by using two assessment tools. The SPIKES competence form,^28^ with 14 items, assessed the participants’ compliance with the SPIKES protocol. Each item was scored as “yes” or “no,” resulting in an overall score (range 0–14). The experts determined a cut-off score using the modified Angoff method.^45^ A passing score was 11 and above, and a failing score was below 11. We used the modified Breaking Bad News Assessment Schedule (mBAS) to evaluate communication.^46^ Rather than allocating points proportionally according to the results obtained, the mBAS is reversed, going from 1 (very good) to 5 (very poor). Overall scores ranged from 5–25. A passing score of 14 or lower was also set by the experts. A failing score was above 14.

Assessments were made in two rounds. In the first round, raters independently rated the video. If items were adjacent raw disagreements between raters (more than a one-point difference), they watched the video together, discussed it, and scored it again.

### Statistical Analysis

The investigators entered the data collected into the R software, version 3.4.1 (the R Foundation). The statistician used SAS version 9.4 (Cary, NC). We compared the homogeneity of the CG and of the TG at pre-test with χ2 test and Fisher’s exact test for qualitative variables and with the Mann-Whitney U test for quantitative parameters.

A generalized linear mixed model^47^ (GLMM) measured changes before and after the BBNSBT in self-efficacy, the SPIKES competence form and the mBAS. We adjusted the effects of time, group, and group-by-time by the study year as a confounding factor. GLMMs were performed with a covariance matrix of the compound symmetry type.^48^ We performed the McNemar’s test to compare the proportion of students who passed the SPIKES competence form and the mBAS cut-offs between pre-test and post-test within the groups.

Furthermore, two further analyses were considered. First, we calculated the relative gains between pre-and post-test within the two groups by means of the following formula: [(post-test – pre-test) / pre-test]. A Mann-Withney U test was used to compare relative gains. Second, we tested whether the BBNSBT could help fill the performance gap between participants with limited clinical experience (less than one year) in the TG and participants with clinical experience (more than one year) in the CG by means of a Mann-Whitney U test. Results were considered statistically significant at the 5% critical level (p< 0.05).

## RESULTS

### Participants’ Sociodemographic Data

Table 1 summarizes the sociodemographic parameters for gender, age, BBN experience, study year, training before BBN simulation training, and pre-test assessment scores. No statistically significant difference was found between the TG and the CG for gender (p = 0.24) and BBN experience (p = 0.44). No participants had attended a communication skills training workshop before the BBNSBT. A statistically significant difference was found for study years (p<0.001). In the CG, participants were predominantly in the third or fourth year of medical school whereas in the TG, they were predominantly in their second year. There was also a statistically significant difference in mean age (p = 0.02), although all students were between 22 and 26 years old.

### Pre-test Assessment

Pre-test assessment results are reported in Table 1. We found no statistically significant difference between the TG and the CG with regard to self-efficacy (p = 0.74). However, at baseline the CG had better scores in the SPIKES Competence Form (p = 0.03) and for BBN skills according to the mBAS (p = 0.02).

### Post-test Assessment

Table 2 presents the results at pre-test and post-test for each group, time effect, group effect, and group-by-time effect. These effects were adjusted by study year. There was a significant group-by-time effect of the training on participants’ self-efficacy (p<0.001). Self-efficacy improved significantly over time, with a 55% enhancement for the TG (p<0.001), while it fell slightly in the CG (2.6% reduction; p = 0.5) (Table 3). The difference between these gains in the two groups was highly significant (p<0.001).

A significant group-by-time effect (p<0.001) of the training on the BBN process was also found for the SPIKES competence form. There was a 33.3% improvement (p<0.001) between pre-test and post-test in the TG while there was no significant gain for the CG. The difference between these gains in the groups was statistically significant (p<0.001). With regard to the measurement of communication skills with the mBAS during BBN, we found a significant group-by-time effect (p<0.001) of the training on communication skills. There was a 23.53% reduction in the non-effective communication skills of the TG participants, but 0% in the CG. The difference between the groups was statistically significant (p<0.001).

### Cut-off Scores

Table 4 shows the proportion of students deemed competent when cut-off scores were applied to the SPIKES and the mBAS between groups at pre-test and post-test, as well as between the times within the same group. There was no statistically significant difference at pre-test between the CG and TG for the level of either SPIKES-competent students (CG 15/31, 48.4%; and TG 11/39, 29.7%) or mBAS-competent students (CG 10/31, 32.3%; and TG 4/37, 10.8).

At post-test, we found a statistically significant (p = 0.02) difference for the SPIKES cut-off score: the TG had a higher number of participants passing the cut-off score (27 students passed; 73.0%) than the CG (14 passed; 45.2%). More TG students (23 of the 37; 62.2%) passed the mBAS cut-off score than CG students (11 of the 31; 35.5%), but without a statistically significant difference (p = 0.07). While there was a statistically significant improvement in pre- and post-test scores for the TG in SPIKES and the mBAS (p<0.001 for both), we didn’t find a significant change in the CG scores (SPIKES: p = 0.92; mBAS: p=0.74).

### Clinically Inexperienced Participants Benefiting from Simulation Vs Clinically Experienced with No Simulation Sessions

Further analysis compared participants with limited clinical experience (less than one year) in the TG (n=25) and participants with clinical experience (more than one year) in the CG (n=23) (Table 5). We found no statistically significant difference between subgroups with respect to perceived self-efficacy in pre-test (p=0.13). In post-test, the difference between the two subgroups was highly significant (p<0.001). The self-efficacy of students with limited clinical experience benefiting from simulation was higher than in the more experienced group. While BBN skills were statistically higher in students with clinical experience in the CG at pre-test, (p=0.049), we observed no differences post-test in the two subgroups (p=0.34). The results showed that participants with limited clinical experience made up the difference. Analyses showed the same for communication skills during BBN.

## DISCUSSION

Medical educators aim to identify the best methods to prepare students for clinical practice. Traditional training is the common pedagogical method for learning clinical skills.^49^ Trainees rarely learn BBN in real clinical practice due to the paucity of opportunities^32^,^50^ and the fact that clinical preceptors are rarely available to give feedback.^3^,^6^,^32^,^50^ At pre-test, our study shows a low level of participant experience and a lack of BBN skills, especially in the TG. Chiniara et al.^51^ define the “simulation zone” as areas in which simulation education may be better suited than other methods. BBN is an example of the HALO quadrant: high impact on the patient and low opportunity to practice.

This feasibility study assessed the impact of a four-hour ED BBNSBT compared to clinical internship. It was hypothesized that BBNSBT would have the potential to increase participant self-efficacy in BBN communication and management, adherence to BBN stages and processes, and to improve communication skills during BBN. Our results revealed that this training increased self-efficacy perception. Participants had a low level of self-efficacy in pre-test. After the BBNSBT, the TG reported being more confident about their knowledge and application of BBN and about their ability to perform BBN compared to the CG. This confirms the results of another, smaller study (n = 20), which showed an improvement in confidence and self-efficacy.^52^

These findings may be explained by Bandura’s social cognitive theory,^53^ which suggests four ways to enhance self-efficacy that we identify in the BBNSBT: 1) enactive attainment (performing the action during the role-play simulation); 2) vicarious experience (observing the video and watching other participants in the role-plays); 3) verbal persuasion (facilitators support the students during the debriefings); and 4) psychological safety during the simulations. Moreover, the perceived self-efficacy of students in the CG with more clinical experience decreased. This result could have different potential explanations, notably that the pre-test may have led to introspection and reflection about their BBN and communication skills.

Communication with patients and their families is one of the Accreditation Council for Graduate Medical Education Milestones for EM residents, specifically the fourth level of BBN.^54^,^55^ Our research used two validated assessment tools that allow for standardization of the evaluation and training. The results demonstrate that BBNSBT using role-playing and debriefing enhances participant BBN learning and performance compared with the traditional learning paradigm and direct immersion in acute clinical situations. BBNSBT offers the opportunity to teach BBN and communication skills to students and young residents in a psychologically safe environment, preventing harm to patients and family members. It allows each participant to announce bad news and observe several BBN simulations with debriefings.

By contrast, in the traditional curriculum role modeling at the bedside could have a negative impact on patients and relatives when medical students or residents engage in inappropriate communication behaviors,^56^,^57^ such as not keeping patients or family members adequately informed or using medical words they do not understand. More students in the TG reached the cut-off scores: 73% for SPIKES and 62.2% for the mBAS vs 45.2% and 35.5% in the CG. These results demonstrate the relevance of BBNSBT in communicating bad news in the ED. However, the difference between the groups for the mBAS cut-off score is not significant. BBNSBT probably focuses more on SPIKES than on communication behavior. It may be necessary to create an advanced course centered on communication skills rather than on SPIKES.

Despite this, BBNSBT offers experiential learning for participants. From the simulation experience, the debriefing process leads students to explore their frames, incorporate new frames such as SPIKES skills, and re-practice these new skills. This process allows knowledge to be acquired through experience.^56^ Moreover, participants had access to ED BBN experts for four hours, which, unfortunately, is unlikely to happen in real clinical practice.

Additional data analyses allowed us to address a new question: Is BBNSBT more useful for students with less than one year of clinical experience? We found a statistically significant difference in the pre-test. Students with limited clinical experience reached the same level of BBN skills as students with more clinical experience after the BBNSBT. The gap between these groups could be filled by simulation training, without the pitfalls of stress and discomfort of direct clinical exposure. No study has previously focused on this question. In fact, BBNSBT used a step–by-step process involving novice participants to bring them to a higher level. The first step involved theoretical explanations given via video, discussions, and lectures. Each simulation, and especially each debriefing, further enhanced the participants’ skills.

One strength of the study is that we paid special attention to the theoretical background upon which the training and evaluation were based, using the widespread SPIKES^28^ theoretical model and the INACSL Standards of Best Practice for Simulation^SM^.^41^,^42^ Moreover, the simulations were well designed, the debriefings were standardized, and the facilitators were trained and experienced. We believe that it is mandatory to meet the INACSL Standards of Best Practice, as well as work with simulation experts to obtain positive results with simulation training.

The next steps for research and pedagogical method improvement can be identified based on these results. Further research is needed to investigate the role of an advanced course in BBN. As BBN is not a required skill for EPs, it would be interesting to investigate whether BBNSBT is feasible and effective in other areas such as obstetrics, intensive care units, etc. Finally, we think that e-learning preparation before BBNSBT, as described for a training on managing low urine output,^58^ could replace some of the in-person time.

## LIMITATIONS

According to Kirkpatrick’s four-level training evaluation model,^59^ the self-efficacy and skills assessment used in the simulation are categorized at level 2, which is a low level.^60^ Moreover, we assessed the impact of BBNSBT just after training. Skills transfer to a real clinical setting is not guaranteed and does not allow for any definite conclusion with regard to the actual impact on patients or family members.^50^ Future studies could assess the impact of this training in the workplace and on skills retention over time.^60^ Despite these limitations, the results are very encouraging given that training is only four hours long, a significantly shorter period than other programs previously described.^25^

While the three scenarios used for the assessments share similarities, they were different before and after the training, as in real life. Cluster randomization resulted in an inequitable distribution of participants. Despite this heterogeneity, statistical analyses adjusted by study year seem to prove that BBN training has an impact on students. This study assessed the impact of a four-hour BBN training, but we cannot be sure that this duration would be more effective than two or six hours. Finally, we did not assess the emotional impact of BBNSBT and BBN assessment on trainees. It would be interesting to know whether BBNSBT elicits a different response than traditional internships.

## CONCLUSION

Training programs aspire to produce competent emergency physicians including excellence in the domains of professionalism and communication. According to the EM Milestones, the target for a trainee ready to graduate for “patient-centered communication (ICS1)” specifically includes being able to deliver bad news. The results of this study revealed that a short, simulation-based training with a debriefing session may improve the self-efficacy, BBN skills, and communication skills of medical students and young residents in the ED. Role-playing appears to be an effective and feasible way for trainees to master BBN and acquire patient-centered skills. Further studies should assess the transfer and retention of these skills as well as when to implement the simulation training in the curriculum.

## Supplementary Information



## Figures and Tables

**Table 1:** Sociodemographic characteristics and pre-test assessment scores by group.

Parameters		Control group (n=31)	Training group (n=37)	P-value
Age (years)	Median (Q1–Q3)	24 (23–26)	23 (22–25)	0.02^1^
BBN experiencxe	n (%)			0.44^2^
None		23 (74.2%)	31 (83.8%)	
Occasional (1–2 times a week)		7 (22.6%)	6 (16.2%)	
Frequent (4–5 times a week)		1 (3.2%)	0	
Study year^3^	n (%)			< 0.001^2^
Second-year medical student		8 (25.8%)	25 (67.6%)	
Third-year medical student		14 (45.2%)	3 (8.1%)	
Fourth-year medical student		5 (16.1%)	4 (10.8%)	
EM resident		4 (12.9%)	5 (13.5%)	
Training before BBN	n (%)			
No		31 (100%)	37 (100%)	
Self-efficacy	Median (Q1–Q3)	1.50 (0.79–1.85)	1.46 (0.96 – 2.00)	0.74^1^
SPIKES competence form	Median (Q1–Q3)	10 (9–12)	8 (7–11)	0.03^1^
mBAS	Median (Q1–Q3)	15 (14–18)	17 (16–18)	0.02^1^

*BBN*, breaking bad news; *mBAS*, modified Breaking Bad News Assessment Schedule: items are reversed, from 1 (very good) to 5 (very poor); *SPIKES*, Setting, Perception, Invitation, Knowledge, Emotions and Summary; *EM*, emergency medicine.

1 Mann-Whitney U test;

2 Fisher’s exact test;

3 Study year is classified by increasing order: the lowest level is second year and the highest is emergency medicine resident.

**Table 2:** Training effects on self-efficacy, the SPIKES competence form and the mBAS: time effect, group effect and group-by-time effect for the control group and the training group.

Parameters	Pre-test	Post-test	Time effect p-value^1^	Group effect p-value^1^	Group-by-time effect p-value^1^
Self-efficacy					
CG (n=31)	1.43±0.64	1.32±0.71	0.37	0.62	< 0.001
TG (n=37)	1.51±0.66	2.40±0.60			
SPIKES Competence Form					
CG (n=31)	9.97±2.66	9.93±2.93	< 0.001	0.8	< 0.001
TG (n=37)	8.62±2.55	11.54±2.13			
mBAS					
CG (n=31)	15.58±2.95	15.42±2.88	< 0.001	0.96	< 0.001
TG (n=37)	17.24±2.42	13.7±2.77			

*mBAS*, modified Breaking Bad News Assessment Schedule: items are reversed, from 1 (very good) to 5 (very poor); *SPIKES*, Setting, Perception, Invitation, Knowledge, Emotions and Summary; *CG*, control group; *TG*, training group.

1 Adjusted by study year.

**Table 3:** Relative gains between pre-test and post-test for the control group and the training group.

Parameters	Median	IQR	P-value^1^
Self-efficacy			
CG (n=31)	−2.6	−36.5–9.22	< 0.001
TG (n=37)	55.6	24.78–148.41	
SPIKES Competence Form			
CG (n=31)	0	−22.5–28.64	< 0.001
TG (n=37)	33.3	16.67–71.43	
mBAS			
CG (n=31)	0	−14.17–15.76	< 0.001
TG (n=37)	−23.53	−31.25–5.88	

*IQR*, interquartile range; *mBAS*, modified Breaking Bad News Assessment Schedule; *SPIKES*, Setting, Perception, Invitation, Knowledge, Emotions and Summary; *CG*, control group; *TG*, training group.

1 Mann-Whitney U test.

**Table 4:** Cut-off scores for SPIKES and the mBAS for the control group and the training group, at pre-test and post-test.

Parameters	CG (n=31)	TG (n=37)	P-value
SPIKES cut-off pre-test			
Failed	16 (51.6%)	26 (70.3%)	0.11^1^
Passed	15 (48.4%)	11 (29.7%)	
SPIKES cut-off post-test			
Failed	17 (54.8%)	10 (27.0%)	0.02^1^
Passed	14 (45.2%)	27 (73.0%)	
Comparison of the success rate within groups (P-value^3^) mBAS cut-off pre-test	0.92	< 0.001	
Failed	21 (67.7%)	33 (89.2%)	0.07^2^
Passed	10 (32.3%)	4 (10.8%)	
mBAS cut-off post-test			
Failed	20 (64.5%)	14 (37.8%)	0.07^1^
Passed	11 (35.5%)	23 (62.2%)	
Comparison of the success rate within groups (P-value^3^)	0.74	< 0.001	

*mBAS*, modified Breaking Bad News Assessment Schedule; *SPIKES*, Setting, Perception, Invitation, Knowledge, Emotions and Summary; *CG*, control group; *TG*, training group.

1 X^2^ test;

2 Fisher’s exact test;

3 McNemar test.

**Table 5:** Comparison of the results of students with limited clinical experience in the training group and students with more than one year of clinical experience in the control group.

	Pre-test			Post-test		
	
Parameters	Median	IQR	P-value^1^	Median	IQR	P-value^1^
	
Self-efficacy						
Clinical apprenticeship > 1 year (n = 23)	1.71	1.01–1.98	0.13	1.18	0.84–2.12	0.001
Limited clinical apprenticeship (n = 25)	1.29	0.83–1.67		2.14	1.88–2.5	
SPIKES						
Clinical apprenticeship > 1 year (n = 23)	10	9.5–12	0.049	11	8–13	0.34
Limited clinical apprenticeship (n = 25)	8	7–11		12	10–13	
mBAS						
Clinical apprenticeship > 1 year (n = 23)	15	13–18	0.02	15	13–17	0.4
Limited clinical apprenticeship (n = 25)	17	17–19		14	12–16	

*IQR*, interquartile range; *SPIKES*, Setting, Perception, Invitation, Knowledge, Emotions and Summary; *mBAS*, modified Breaking Bad News Assessment Schedule.

1 Mann-Whitney U test.
