# Supplementary material for: Efficacy of a Short Role-Play Training on Breaking Bad News in the Emergency Department
Source: West J Emerg Med. 2019 Oct 14;20(6):893–902. doi: 10.5811/westjem.2019.8.43441 (PMC6860397; doi:10.5811/westjem.2019.8.43441)
Supplement: Supplementary file 1 [file wjem-20-893-s001.docx]

**En général**

Pour chaque objectif, estimez, selon vous, votre niveau de connaissance, de capacité d’action, et votre motivation à appliquer ces connaissances/compétences dans votre activité professionnelle **:**

1. Annoncer une mauvaise nouvelle

**J’ai des connaissances**

pour annoncer une mauvaise nouvelle

| 0 | 1 | 2 | 3 | 4 | 5 |
| --- | --- | --- | --- | --- | --- |
| **Pas du tout** | **Très peu** | **Un peu** | **Assez** | **Beaucoup** | **Énormément** |

Pour quelles raisons ?

**Je me sens capable**

d’annoncer une mauvaise nouvelle

| 0 | 1 | 2 | 3 | 4 | 5 |
| --- | --- | --- | --- | --- | --- |
| **Pas du tout** | **Très peu** | **Un peu** | **Assez** | **Beaucoup** | **Énormément** |

Pour quelles raisons ?

**J’appliquerai ces connaissances/compétences**  dans ma pratique professionnelle

| SO | 0 | 1 | 2 | 3 | 4 | 5 |
| --- | --- | --- | --- | --- | --- | --- |
| **Sans objet** | **Pas du tout** | **Très peu** | **Un peu** | **Assez** | **Beaucoup** | **Énormément** |

Pour quelles raisons ?

……………………………………………………………………………………………………………………………………………………………………………………………………………………………………………………………………………………..……………………………………………………………………………………………………………………………………………………………………………………………………………………………………………………………………………………………………………………………..……………………………………………………………………………………………………………………………………………………………………………………………………………………………………………………………………………………..………………………………………………………………………………

1. Gérer votre communication verbale lors de l’annonce de mauvaise nouvelle

**J’ai des connaissances**

pour bien communiquer verbalement

| 0 | 1 | 2 | 3 | 4 | 5 |
| --- | --- | --- | --- | --- | --- |
| **Pas du tout** | **Très peu** | **Un peu** | **Assez** | **Beaucoup** | **Énormément** |

**Je me sens capable**

de gérer ma communication verbale

| 0 | 1 | 2 | 3 | 4 | 5 |
| --- | --- | --- | --- | --- | --- |
| **Pas du tout** | **Très peu** | **Un peu** | **Assez** | **Beaucoup** | **Énormément** |

**J’appliquerai ces connaissances/compétences**  dans ma pratique professionnelle

| SO | 0 | 1 | 2 | 3 | 4 | 5 |
| --- | --- | --- | --- | --- | --- | --- |
| **Sans objet** | **Pas du tout** | **Très peu** | **Un peu** | **Assez** | **Beaucoup** | **Énormément** |

1. Gérer votre communication non-verbale et vos émotions lors de l’annonce de mauvaise nouvelle

**J’ai des connaissances**

pour bien communiquer de manière non-verbale et gérer mes émotions

| 0 | 1 | 2 | 3 | 4 | 5 |
| --- | --- | --- | --- | --- | --- |
| **Pas du tout** | **Très peu** | **Un peu** | **Assez** | **Beaucoup** | **Énormément** |

**Je me sens capable**

de gérer ma communication non-verbale et mes émotions

| 0 | 1 | 2 | 3 | 4 | 5 |
| --- | --- | --- | --- | --- | --- |
| **Pas du tout** | **Très peu** | **Un peu** | **Assez** | **Beaucoup** | **Énormément** |

**J’appliquerai ces connaissances/compétences**  dans ma pratique professionnelle

| SO | 0 | 1 | 2 | 3 | 4 | 5 |
| --- | --- | --- | --- | --- | --- | --- |
| **Sans objet** | **Pas du tout** | **Très peu** | **Un peu** | **Assez** | **Beaucoup** | **Énormément** |

1. Identifier les émotions et les réactions d’autrui lors de l’annonce de mauvaise nouvelle

**J’ai des connaissances**

pour identifier les réactions de mon interlocuteur et y réagir adéquatement

| 0 | 1 | 2 | 3 | 4 | 5 |
| --- | --- | --- | --- | --- | --- |
| **Pas du tout** | **Très peu** | **Un peu** | **Assez** | **Beaucoup** | **Énormément** |

**Je me sens capable**

d’identifier les réactions de mon interlocuteur et d’y réagir adéquatement

| 0 | 1 | 2 | 3 | 4 | 5 |
| --- | --- | --- | --- | --- | --- |
| **Pas du tout** | **Très peu** | **Un peu** | **Assez** | **Beaucoup** | **Énormément** |

**J’appliquerai ces connaissances/compétences**  dans ma pratique professionnelle

| SO | 0 | 1 | 2 | 3 | 4 | 5 |
| --- | --- | --- | --- | --- | --- | --- |
| **Sans objet** | **Pas du tout** | **Très peu** | **Un peu** | **Assez** | **Beaucoup** | **Énormément** |

1. Accompagner le patient/sa famille dans la gestion de ses émotions

**J’ai des connaissances**

pour accompagner mon interlocuteur dans la gestion de ses émotions

| 0 | 1 | 2 | 3 | 4 | 5 |
| --- | --- | --- | --- | --- | --- |
| **Pas du tout** | **Très peu** | **Un peu** | **Assez** | **Beaucoup** | **Énormément** |

**Je me sens capable**

d’accompagner mon interlocuteur dans la gestion de ses émotions

| 0 | 1 | 2 | 3 | 4 | 5 |
| --- | --- | --- | --- | --- | --- |
| **Pas du tout** | **Très peu** | **Un peu** | **Assez** | **Beaucoup** | **Énormément** |

**J’appliquerai ces connaissances/compétences**  dans ma pratique professionnelle

| SO | 0 | 1 | 2 | 3 | 4 | 5 |
| --- | --- | --- | --- | --- | --- | --- |
| **Sans objet** | **Pas du tout** | **Très peu** | **Un peu** | **Assez** | **Beaucoup** | **Énormément** |

1. Mener un entretien d’annonce de mauvaise nouvelle structuré

**J’ai des connaissances**

pour mener un entretien structuré

| 0 | 1 | 2 | 3 | 4 | 5 |
| --- | --- | --- | --- | --- | --- |
| **Pas du tout** | **Très peu** | **Un peu** | **Assez** | **Beaucoup** | **Énormément** |

**Je me sens capable**

de mener un entretien structuré

| 0 | 1 | 2 | 3 | 4 | 5 |
| --- | --- | --- | --- | --- | --- |
| **Pas du tout** | **Très peu** | **Un peu** | **Assez** | **Beaucoup** | **Énormément** |

**J’appliquerai ces connaissances/compétences**  dans ma pratique professionnelle

| SO | 0 | 1 | 2 | 3 | 4 | 5 |
| --- | --- | --- | --- | --- | --- | --- |
| **Sans objet** | **Pas du tout** | **Très peu** | **Un peu** | **Assez** | **Beaucoup** | **Énormément** |

1. Faire passer un message difficile en limitant autant que possible les dommages pour le récepteur

**J’ai des connaissances**

pour annoncer une mauvaise

nouvelle en limitant les dommages pour le récepteur

| 0 | 1 | 2 | 3 | 4 | 5 |
| --- | --- | --- | --- | --- | --- |
| **Pas du tout** | **Très peu** | **Un peu** | **Assez** | **Beaucoup** | **Énormément** |

**Je me sens capable**

d’annoncer une mauvaise nouvelle en limitant les dommages pour le récepteur

| 0 | 1 | 2 | 3 | 4 | 5 |
| --- | --- | --- | --- | --- | --- |
| **Pas du tout** | **Très peu** | **Un peu** | **Assez** | **Beaucoup** | **Énormément** |

**J’appliquerai ces connaissances/compétences**  dans ma pratique professionnelle

| SO | 0 | 1 | 2 | 3 | 4 | 5 |
| --- | --- | --- | --- | --- | --- | --- |
| **Sans objet** | **Pas du tout** | **Très peu** | **Un peu** | **Assez** | **Beaucoup** | **Énormément** |
